# An Account of Two Cases of Hæmaturia

**Published:** 1783

**Authors:** 


### SECTION II.

### Essays and Observations.

I. *An Account of two cafes of Hæmaturia. By* Robert Bland, M. D. *Phyfician-man-midwife to the Weftminfter General Difpenfary.*

A. E. a girl twelve years old, pale, thin, and of an irritable temper and habit, attempted about two years ago to lift a weight much beyond her ftrength. On the fecond or third day after this, blood was obferved in her urine, but without having been preceded by any remarkable pain. The quantity at firft was not confiderable; perhaps not exceeding an ounce in twenty-four hours. It feemed to yield, in fome degree at leaft, to a cooling regimen and the occafional ufe of gentle purgatives. But altho' by purfuing this method, the quantity of blood that came with the urine feemed to be diminifhed, and and ſometimes an interval of two or three days was obtained in which no blood was diſcharged, yet upon any agitation of the paſſions, which it was not always poſſible to guard againſt, the diſcharge returned with increaſed violence. Upon theſe occaſions glyſters of linſeed oil, which has been thought to have a ſpecific property of reſtraining internal hæmorrhages, were injected, and the ſame medicine combined with a mucilage of gum arabic given by the mouth. To theſe were occaſionally added nitre, cream of tartar, &c.

By perſiſting in this method, but particularly by guarding againſt exceſs of exerciſe or paſſion, a ceſſation of the diſcharge was at length obtained for ſeveral weeks, after which it returned again with the addition of a new ſymptom, viz. a pain on the left ſide, a little above her hip and inclining backwards, ſeemingly in the direction of the ureter. This has ſince been a concomitant, or rather as the friends of the child think, a forerunner of the diſeaſe. This pain, they have obſerved, conſtantly increaſes as the Sun riſes to its meridian, and that whether the child is walking out or confined at home. This increaſed pain never fails to be followed by an increaſed diſcharge of blood.

 Three

Three or four ounces of blood were now taken away, and recourſe was had to the ſame method that ſeemed to have been ſucceſsful before, with the addition of decoction of bark and elixir of vitriol. After ſeveral months continuance in a greater or leſs degree, the diſcharge once more ſtopped for the ſpace of three months, but at the end of that time returned again in a greater degree than ever, and was preceded by the ſame pain as before. During the laſt five months it has again entirely ceaſed.

She has never loſt her appetite. Her ſtrength and fleſh are increaſed, and ſhe appears to have loſt that diſpoſition to fever, which from a continuance of ſeven or eight years, ſeemed to have been conſtitutional. During the latter part of laſt ſummer ſhe uſed cold bathing, without any material effect upon the diſcharge, but ſeemingly with advantage to her general health.

As diſeaſes of this kind are not of frequent occurrence, I think it not improper to add the following brief account of the caſe of a gentleman, eminent in the law, which he related to me a few days ſince.

About three years ago, without any previous indiſpoſition, or apparent cauſe, he obſerved blood to be diſcharged with his urine. As he was alarmed at the appearance, he conſulted

ſome

ſome of the moſt eminent phyſicians and ſurgeons in this metropolis, and conformed to ſuch regimen, and took ſuch medicines, chiefly of the corroborant and aſtringent claſſes, as they adviſed, for a conſiderable time, without any material effect, except that by long and rigorous abſtinence he had reduced his ſtrength, and impaired his general health. He was once tempted to believe that the gout, to which he had been ſubject ſeveral years, was kept away by it; but time proved the fallacy of that hope. One day, after riding, he felt a moſt excruciating pain in his loins, which obliged him to go double. This continued ſeveral minutes, when he ſuddenly felt ſomething drop into his bladder, which was followed by immediate eaſe. Soon after he had occaſion to make water, when a clot of blood of the ſize of a pea was ſeen in his urine. From this time he laid aſide all medicines, and continued to have the diſcharge in a greater or leſs degree according as his exerciſe was more or leſs violent, or his mind more or leſs agitated. About the month of June laſt, being excited by ſome unexpected ſucceſs in a buſineſs he had deemed hopeleſs, he indulged in a more liberal doſe of wine than he had now for many months taken, ſo as to be intoxicated.

The

The next morning he obferved that the urine he had paffed in the night was free from blood, and he has had no return of the difcharge.

*St. Alban's Street,*

*June* 1, 1783.

